# The role of antimalarials in lupus nephritis: a review

**DOI:** 10.1186/1546-0096-10-S1-A29

**Published:** 2012-07-13

**Authors:** Senq-J Lee, Earl D Silverman, Joanne M Bargman

**Affiliations:** 1 The Hospital for Sick Children, Toronto, ON, Canada; 2 Toronto General Hospital, Toronto, ON, Canada

## Purpose

Systemic lupus erythematosus (SLE) is a chronic multisystem autoimmune disease affecting various organs, with lupus nephritis being one of the most important and common serious manifestations. Antimalarials (AM) are one of the many immunomodifying medications used in SLE, however less known is its role in lupus nephritis. Our study examined the history of AM use, theorized mechanisms of action, efficacy in SLE, in particular in lupus nephritis, safety in pregnancy, and overall safety profile.

## Methods

We conducted a search of all relevant literature using Medline (OVID and EMBASE) and PubMed. We included randomized-controlled trials, observational cohort studies, and case-control studies. Case reports were only included for the adverse effect profile of AM.

## Results

•AM use benefits patients with SLE including improving survival, reducing disease activity, new organ involvement, integument damage, risk of infection, risk of thrombosis, and possible cardioprotective and anti-malignancy effects.

**Figure 1:**
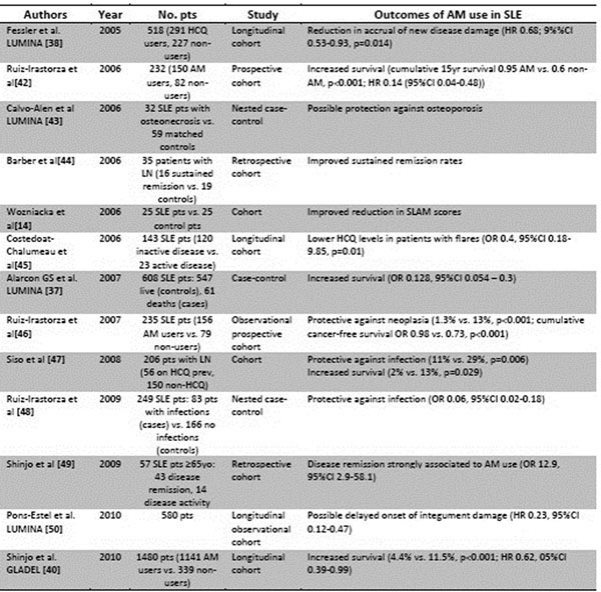
Overview of effects of antimalarials on lupus disease and activity – articles from 2005 to 2010. LN=lupus nephritis.

**Figure 2:**
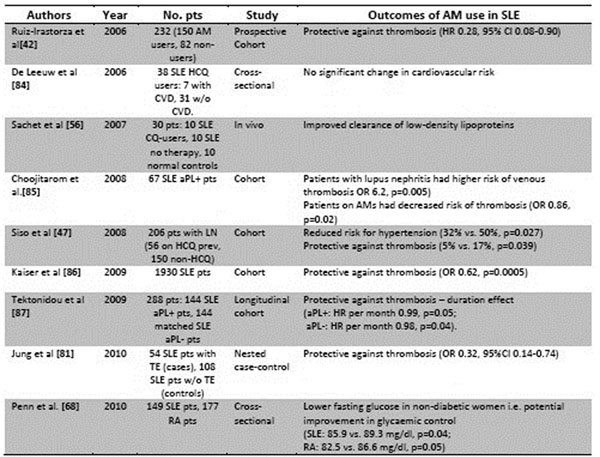
Overview of effects of antimalarials on cardiovascular disease and thrombosis – articles from 2005-2010. CVD = cardiovascular disease. aPL = antiphospholipid antibodies. TE = thromboembolic events. RA = rheumatoid arthritis.

•In lupus nephritis, AM use improves time to end-stage renal disease, disease activity, flare rates, disease remission as an adjunct with other immunomodifying drugs, and reduced cumulative corticosteroid use.

**Figure 3:**
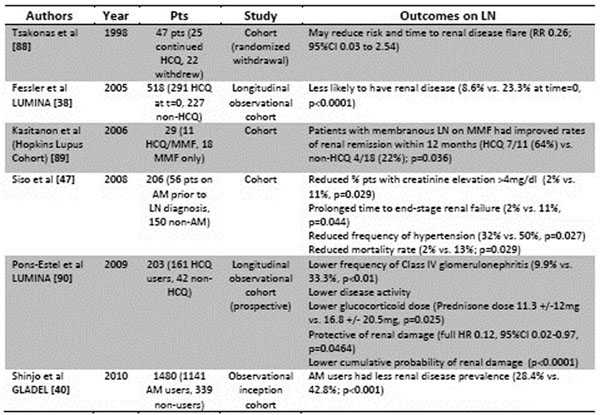
Overview of studies on AMs and outcomes related to LN. MMF = mycophenolate mofetil.

•AM are safe to use and should be continued in pregnant SLE patients for its beneficial effects of reducing disease activity, flare rates, cumulative corticosteroid requirements, and possible reduction in development of cardiac neonatal lupus erythematosus.•AM have a good safety profile, with gastrointestinal symptoms being the most common. Careful regular monitoring for retinopathy is recommended as per American Academy of Ophthalmology.

**Figure 4:**
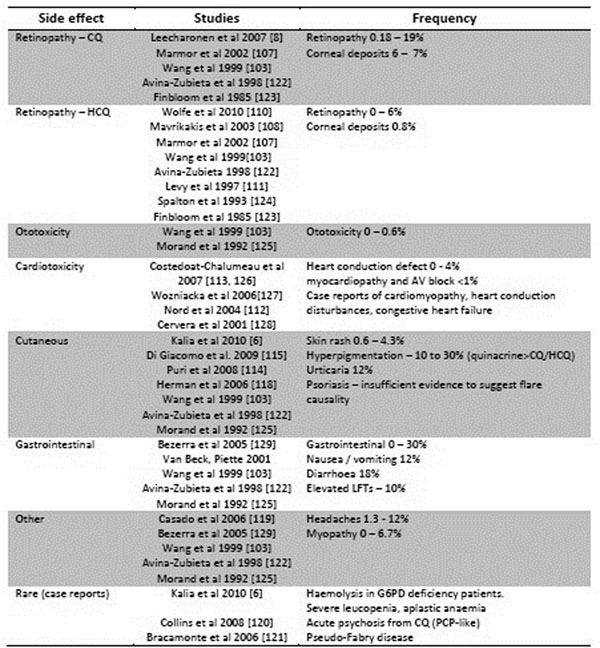
Overview of AM potential toxicity/adverse events

•In patients with renal disease, caution with dosing and careful monitoring for adverse events should be taken.

## Conclusion

AM are medications which confer many benefits to patients with SLE and lupus nephritis, with a good safety profile.

## Disclosure

Senq-J. Lee: None; Earl D. Silverman: None; Joanne M. Bargman: None.

